# Predictors of atrial fibrillation early recurrence following cryoballoon ablation of pulmonary veins using statistical assessment and machine learning algorithms

**DOI:** 10.1007/s00380-018-1244-z

**Published:** 2018-08-23

**Authors:** Jan Budzianowski, Jarosław Hiczkiewicz, Paweł Burchardt, Konrad Pieszko, Janusz Rzeźniczak, Paweł Budzianowski, Katarzyna Korybalska

**Affiliations:** 1 Department of Cardiology, Nowa Sól Multidisciplinary Hospital, Nowa Sól, Poland; 2 Faculty of Medicine and Health Sciences, University of Zielona Góra, Zielona Góra, Poland; 3 Department of Biology and Lipid Disorders, Poznań University of Medical Sciences, Poznań, Poland; 4 Department of Cardiology, J. Struś Hospital, Poznań, Poland; 5 Department of Engineering, University of Cambridge, Cambridge, UK; 6 Department of Pathophysiology, Poznań University of Medical Sciences, Poznań, Poland

**Keywords:** Atrial fibrillation, Cryoballoon ablation, Early recurrence, Biomarkers, Machine learning

## Abstract

Inflammation, oxidative stress, myocardial injury biomarkers and clinical parameters (longer AF duration, left atrial enlargement, the metabolic syndrome) are factors commonly related to AF recurrence. This study aims to assess the predictive value of laboratory and clinical parameters responsible for early recurrence of atrial fibrillation (ERAF) following cryoballoon ablation (CBA) using statistical assessment and machine learning algorithms. This study group comprised 118 consecutive patients (mean age, 62.5 ± 7.8 years; women 36%) with paroxysmal (54.1%) and persistent (45.9%) AF who underwent their first pulmonary vein isolation (PVI) performed by CBA (Arctic Front Advance 2nd generation 28 mm). The biomarker concentrations were measured at baseline and after CBA in a 24-h follow-up. ERAF was defined as at least a 30-s episode of arrhythmia registered by a 24 h-Holter monitor within the 3 months following the procedure. 56 clinical, laboratory and procedural variables were collected from each patient. We used two classification algorithms: support vector machines, gradient boosted tree. The synthetic minority over-sampling technique (SMOTE) was used to provide a balanced training data set. Within a period of 3 months 21 patients (17.8%) experienced ERAF. The statistical analysis indicated that the lowered levels of post-ablation TnT (*p* = 0.043) and CK-MB (*p* = 0.010) with the TnT elevation (*p* = 0.044) were the predictors of ERAF following CBA. In addition, diabetes and statin treatment were significantly associated with ERAF after CBA (*p* < 0.05). The machine learning algorithms confirmed the results obtained in the univariate analysis.

## Introduction

Cryoballoon ablation (CBA) of pulmonary veins, along with radiofrequency ablation (RFCA), is a widely acknowledged method of atrial fibrillation (AF) treatment. Pulmonary vein isolation (PVI) is a crucial element of the AF ablation procedure [1].

In our study, we decided to focus on the clinical significance of early recurrence of atrial fibrillation (ERAF) given that many published studies consider it one of the most frequent factors contributing to the failure of the procedure in the long run. It has been estimated that nearly half of patients experience early arrhythmia recurrence in a 3-month follow-up [2–4]. Such conclusions have been drawn in a substudy of a multicenter randomized STOP AF trial where above half (51.5%) of the patients who had undergone CBA suffered from ERAF after the index-PVI procedure. In this study, 55.6% of patients with ERAF experienced LRAF (*p* < 0.001) [4]. Similar results were obtained in the studies on radiofrequency ablation (RF), which revealed that ERAF affected the majority of patients (35–71%) [5, 6].

Considering the limitations of classical statistical analysis (which applies low dimensional data sets, requires prior assumptions about the underlying relationships between variables and predominantly relies on interference), it was attempted to apply the machine learning (ML) methods to facilitate the process of reaching clinical conclusions. ML primarily aims to build systems that can constantly improve their performance and to obtain a higher predicting value by analysing complex relations which so far have not been exposed in linear statistical analysis. Crucially, ML allows to shift the paradigm from analyzing key factors to creating decision-making aides. Being aware of ML potential, we have paid special attention to the fact that ML algorithms are playing an increasingly important role in cardiology. For example, they are used to make predictions and decisions in many cases of cardiovascular diseases allowing for more individualized and targeted treatment [7].

Consequently, this study aimed to verify whether the laboratory and clinical factors responsible for ERAF after CBA of pulmonary veins are considered of equal significance when examined by ML tools of analysis.

## Patients and methods

### Study population

The study consisted of 118 consecutive patients with paroxysmal and permanent AF who were referred to the department of cardiology in Nowa Sól, Poland from May 2016 till September 2017 to undergo their first pulmonary vein isolation (PVI) performed through CBA (Arctic Front Advance 2nd generation 28 mm) in accordance with international guidelines.

Paroxysmal AF was defined as a self-terminating episode which lasted for up to 7 days while persistent AF occurred if arrhythmia took at least 7 days and could require cardioversion. The inclusion criteria were as follows: age > 18, no PVI attempt. Exclusion criteria included: intracardiac thrombi, myocardial infarction or cardiac surgery in the previous 3 months, autoimmune or inflammatory diseases, chronic renal failure, malignancies and previous AF ablation.

ERAF was marked as at least 30-s symptomatic or asymptomatic atrial tachyarrhythmias (AF, atrial tachycardia [AT] or atrial flutter [AFL]) that occurred after catheter ablation. An episode of ERAF on ECG and Holter monitoring was always documented within the first 3 months after the procedure.

Clinical and demographic parameters included: age, sex and comorbidities such as coronary heart disease, hypertension, thyroid diseases, dyslipidemia, diabetes mellitus, impaired glucose tolerance (IGT), impaired fasting glucose (IFG) and valvular heart disease.

All patients who took part in the study signed a written consent while the study protocol was approved by the Medical Ethics Committee of the Poznań University of Medical Sciences.

### Preprocedural evaluation (echocardiography, CT)

Transthoracic echocardiography (TTE) was performed in compliance with European guidelines before the procedure (iE 33, Philips Medical Systems, MA, USA). Transesophageal echocardiography (TEE) was performed 48 h before ablation to exclude thrombus in the left atrial appendage (Epiq 7, Philips Medical Systems, MA, USA). In addition, computed tomography (CT) was required to scrutinize the LA and PVs anatomy. The LA volume was indicated using biplane disk summation while blood pressure was measured in a supine position.

### Ablation procedure

CBA is a single-shot procedure performed by pressurized cryorefrigerant (liquid nitrous oxide) delivered by cryoconsole. Before the procedure the patients had to continue oral anticoagulation (Warfin, Acenokumarol). Dabigatran and Xarelto were withdrawn 24 h before the procedure depending on the patient’s kidney function. All ablation procedures were performed under conscious sedation. The patients had a groin entry venous route catheter introduction with the transseptal puncture performed by means of a Brockenbrough needle (St. Jude Medical). In addition, a 15 Fr steerable sheath (FlexCath Advance, Medtronic) and an integrated inner-lumen circular mapping catheter (CMC, Achieve™; Medtronic, Inc.) were applied. Each freeze was preceded by an occlusion of pulmonary veins which was verified by an injection of contrast medium.

The application time constituted 240 s. The pulmonary veins potentials were recorded before ablation and then compared with the results obtained after the procedure. The final effect was defined as the lack of all pulmonary veins potentials, which was confirmed by a two-directional blockade in a mapping catheter. In the instance when the pulmonary veins potentials could not be recorded, the PV isolation was verified by withdrawing the catheter and placing it in an ostial position. Additional linear lesions and focal deliveries were not performed and adenosine testing was not used.

CBA was performed with a heparin administration following a transseptal puncture and adjusted to maintain the activated clotting time (ACT) for at least 300–350 s throughout the procedure. To avoid phrenic nerve injury (PNI) during the right-sided PVs ablation, phrenic nerve function was monitored by diaphragmatic stimulation which paced the ipsilateral phrenic nerve 30 times per min.

### Biomarker analysis

Venous blood was drawn from a basilica vein to determine the peripheral blood count and the levels of C-reactive protein (CRP), troponin-T (TnT), creatine kinase (CK), creatine kinase-MB (CK-MB), D-dimer, fibrinogen and INR. All tests were carried out at baseline and 24 h after CBA by means of standard laboratory kits.

In the analysis the peripheral blood count was marked with CELL-DYN Ruby (Abbott Diagnostics, USA). D-dimer, Fibrinogen, INR and aPTT were tested using STA-Compact Max (Stago, USA). TnT was marked with a Cobas 6000 device (Roche Diagnostics GmbH) with a cut-off value 14 pg/l. Creatinine, potassium, sodium, transaminases, CRP, CK and CK-MB concentrations were analysed by means of photometric Test (Roche Diagnostics GmbH). Normal reference ranges were as follows: WBC, 4.0–10.0 (× 10^9^/l), Fibrinogen 200–400 mg/dl; CK 0-190 U/l and CK-MB 7–25 U/l. The CRP cut-off value was 0.5 mg/dl while the D-dimer cut-off value equalled 0.5 µg/ml. The extent of biomarker elevation was defined as the post-procedure recorded value minus the baseline value (day 0).

### Post-ablation management and follow-up

The patients were monitored for the first 24 h after CBA. TTE was performed to exclude pericardial effusion. A follow-up examination was scheduled at the outpatient clinic within 3 months when a 24 h-Holter monitoring was carried out. Additional ECG was performed when the patient was symptomatic.

Oral anticoagulants (OA) and AADs were continued for 2–3 months after CBA. AADs were continued in case of atrial tachyarrhythmia recurrence after 3 months following the procedure. The decision regarding the continuation of OA was based on the patient’s individual stroke risk determined by CHA_2_DS_2_-VASc score.

### Statistical analysis

Normality was verified by the Shapiro–Wilk test. Normally distributed variables were compared by means of the Student’s *t* test for two independent and dependent variables. The Mann–Whitney test was used for two independent variables not normally distributed while the Sign test and Wilcoxon matched pairs test were used for two dependent variables. Based on the results from univariate analysis, we performed multivariate analysis using a logistic regression model. McNemar’s test was used on the paired nominal data with contingency tables. Quantitative variables were expressed as a mean and standard deviation or median and interquartile ranges for normal and non-normal distribution of variables, respectively. A *p* value < 0.05 indicated the statistical significance.

The multivariate analysis was performed to identify the predictors of ERAF. Each analysis was performed using STATISTICA 8.0 software (Statsoft, USA).

### Machine learning algorithm in ERAF prediction model

To gain more insight into the issue broached here, we can reformulate the problem of finding relevant factors triggering ERAF to predict the probability of patients experiencing ERAF. ML algorithms allow for obtaining probability predictions of ERAF for individual patients. They also evaluate the significance of individual factors. Thus, two classification algorithms were used for predicting the probability of ERAF: support vector machines (SVM), gradient boosted trees [8, 9]. Both of these models were trained in a supervised fashion, classifying patients to two separate categories. The SVM algorithm was maximizing the gap between two different groups of data-points (patients) while the gradient boosted trees algorithm formed an ensemble of decision trees to gauge which category the analyzed data-point belonged to. The final classifiers were trained on the reduced set of seven variables, which played an important role in predicting ERAF. Due to the imbalanced classes representation, the synthetic minority over-sampling technique (SMOTE) was used to provide a balanced training data set [10]. Higher classification weights for positive examples were added to further improve the sensitivity of the model.

## Results

### Patients characteristics

A total of 118 consecutive patients (37% women, age 62.7 ± 7.8 years, 54.2% with paroxysmal AF) with symptomatic, drug refractory AF were studied. ERAF occurred in 21 (17.8%) patients within the first 3 months after the procedure. A 3-month follow-up was completed in case of all patients. Baseline demographic, clinical and laboratory characteristics have been summarised in Table 1.

**Table 1:** Characteristics of patients at baseline

Characteristics	Total (*n* = 118)
Age (year)	62.7 ± 7.8
Male sex—no. (%)	75 (63.6)
Early recurrences no. (%)	21 (17.8)
Paroxysmal AF	64 (54.2)
Body mass index (kg/m^2^)	32.1 ± 5.0
LA volume (ml)	94.8 ± 34.3
Mean CHA_2_DS_2_-VASC score	2.2 ± 1.1
Mean HAS-BLED score	1.4 ± 0.8
Hypertension—no. (%)	92 (80)
Coronary artery disease = no. (%)	23 (19.5)
Diabetes and prediabetes (IGT + IFG)	27 (22.9)
Dyslipidemia	20 (16.9)
Valvular heart disease	17 (14.4)
Thyroid disease	28 (23.7)
RR syst (mmHg)	128.2 ± 11.9
RR diast (mmHg)	80.1 ± 9.1
RBC (10^6^/ml)	4.8 ± 0.5
Hb (g/dl)	15.2 ± 1.3
Hct (%)	45.1 ± 4.4
MCV (fl)	94.0 ± 4.6
MCH (pg)	31.7 ± 1.7
MCHC (g/dl)	34.3 ± 6.3
RDW-CV (%)	12.7 ± 1.1
WBC (× 10^3^/ml)	7 ± 1.6
Plt (10^3^/ml)	207 ± 51.1
CRP (mg/l)	0.3 ± 0.7
Chol (mg/dl)	179.4 ± 48.9
HDL (mg/dl)	60 ± 14.3
LDL (mg/dl)	111.8 ± 43.2
TG (mg/dl)	126.7 ± 62.7
Glucose (mg/dl)	103.1 ± 18.6
INR	1.6 ± 0.7
aPTTs	39.7 ± 8.6
Fibrinogen (mg/dl)	387.4 ± 76
D-dimers (µg/ml)	0.3 ± 0.4
Troponin T hs (pg/ml)	9.8 ± 6.4
CPK (U/l)	142.9 ± 104.6
CP-MB (U/l)	18.2 ± 9.9
Na (mmol/l)	142.1 ± 2.1
K (mmol/l)	4.5 ± 0,4
Urea (mg/dl)	38.7 ± 9.5
GFR (MDRD)	74.0 ± 15.8
Creatinine (mg/dl)	1.0 ± 0.2
TSH (uIU/ml)	2.2 ± 2.3
AlAT (U/l)	27.7 ± 14
AspAT (U/l)	24.7 ± 6.9
Medication use—no. (%)	
NOAC	82 (69.8)
Beta blockers	104 (88.1)
Statins	64 (54.2)
ACE-I	51 (43.2)
ACE-I + ARB	83 (70.3)
Diuretics	42 (35.6)
Smoking	15 (12.7)

Continuous data are presented as means with SD. Categorical data are presented as counts with their percentage values in brackets

*LV* Left ventricle, *NOAC* novel oral anticoagulants, *ACE-I* angiotensin converting enzyme inhibitor, *ARB* angiotensin II receptor blocker, *CHA*_*2*_*DS*_*2*_*-VASc* (Congestive Heart failure); hypertension; Age ≥ 75 (doubled); Diabetes; Stroke (doubled); Vascular disease; Age 65–74, Sex (female)

The majority of patients had hypertension (80.0%). Diabetes mellitus, coronary artery disease, dyslipidemia, moderate valvular heart disease were present in 22.9%, 19.5%, 16.9% and 14.4% of patients, respectively. The mean CHA_2_DS_2_-VASC score was 2.1 ± 1.1 while the mean HAS-BLED score was 1.4 ± 0.8. Complications related to CBA included: transient phrenic nerve palsy which occurred in nine patients (7.6%) and a thromboembolic event which affected 1 patient (0.8%) in the follow-up.

As shown in Table 2 patients with ERAF had significantly decreased TnT, CK-MB levels, the extent of TnT elevation and RR diastolic pressure following CBA. Lowered INR was noted in patients with ERAF before CBA, which contrasted with those who remained in sinus rhythm (*p* < 0.05).

**Table 2:** Comparison of significant differences between clinical and laboratory characteristics of patients with ERAF and without it following CBA

Characteristics	ERAF (*n* = 21)	Lack of ERAF (*n* = 97)	*p* value
TnT hs (pg/ml) after CBA	816.6 ± 356.9	1024.1 ± 402.4	0.043
TnT hs elevation (pg/ml)	807.5 ± 355.2	1014.2 ± 402.7	0.044
CK-MB after CBA (U/l)	32.0 ± 13.8	41.8 ± 20.02	0.010
RR diast (mmHg) after CBA	71.4 ± 6.6	75.7 ± 9.2	0.039
INR before CBA	1.3 ± 0.5	1.7 ± 0.7	0.012

There was a tendency for a lower fluoroscopy time in patients without ERAF [13.5 ± 5.6 without ERAF vs 15.8 ± 5.4 with ERAF (*p* = 0.08)] which was presented in Table 3.

**Table 3:** Procedure characteristics

	ERAF (*n* = 21)	Lack of ERAF (*n* = 97)	*p* value
Procedural time (min)	106.0 ± 26.7	97.0 ± 26.8	0.169
Fluoroscopy time (min)	15.8 ± 5.4	13.5 ± 5.6	0.091
Cryoenergy application time (min)	30.9 ± 8.0	28.0 ± 9.5	0.089
Number of cryoenergy application	8.4 ± 2.4	7.8 ± 2.7	0.231

Multivariate analysis was performed using logistic regression. The model included CK-MB, TnT following CBA and TnT elevation. The results showed no significant influence of these factors on ERAF.

Comorbidities such as diabetes mellitus and prediabetes (IGT and IFG) were significantly more frequent in patients with ERAF as opposed to patients without it. Patients treated with statins had a more significantly increased risk of ERAF than those who did not receive statins. AADs treatment (amiodarone and propafenone) did not affect ERAF after CBA. Other medications such as beta-blockers, oral anticoagulants, angiotensin converting enzyme inhibitors (ACE-I) and angiotensin II receptor blockers (ARB) were not associated with ERAF.

### Machine learning algorithms

**Fig. 1:**
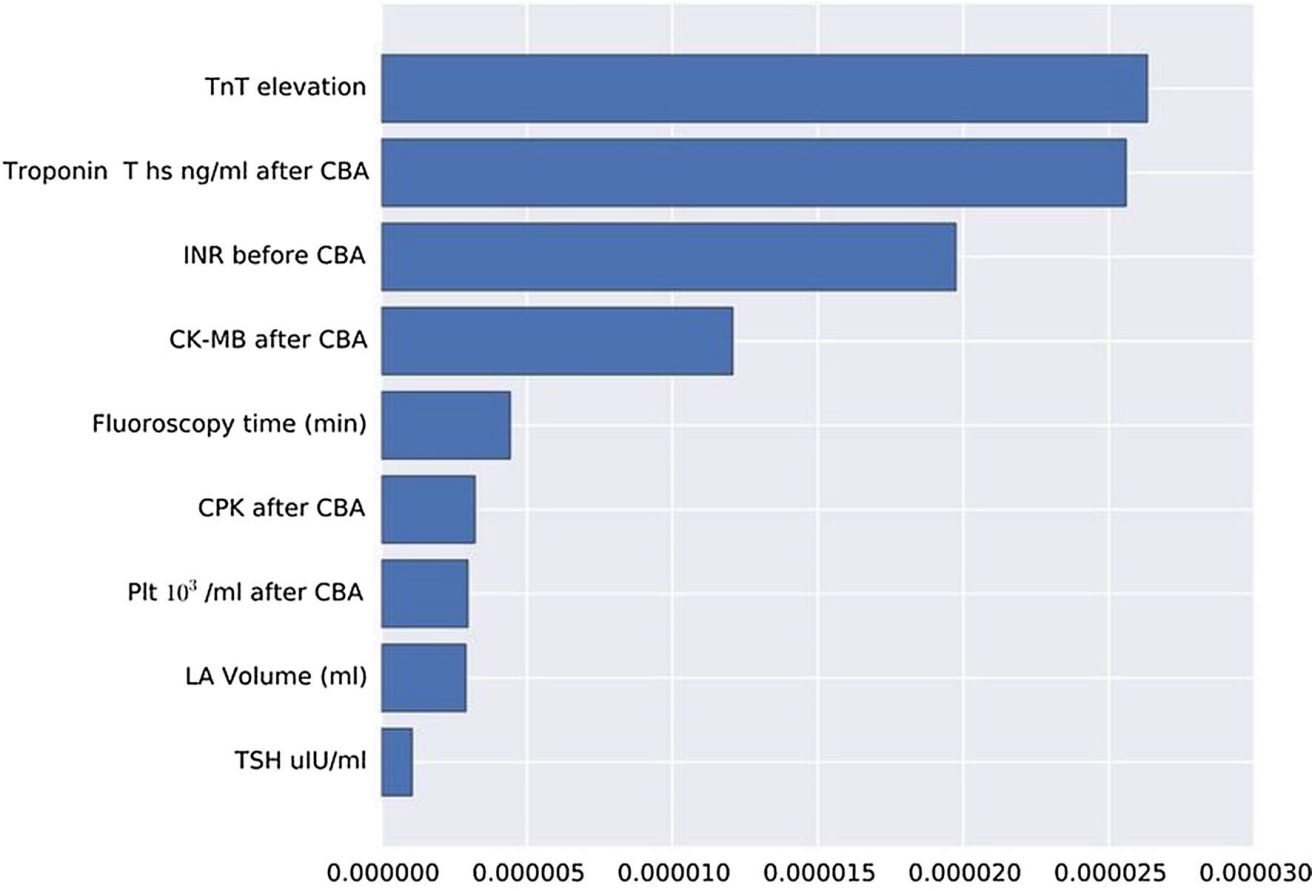
Averaged squared coefficients from the best SVM model with a linear kernel. (page 9)

All of the employed algorithms suffered initially from overfitting due to imbalanced classes. Thanks to weight scaling and over-sampling, gradient boosted random trees and SVM allowed for substantially improving the sensitivity score. Nevertheless, all three models had moderate specificity and sensitivity for predicting ERAF. The best SVM algorithm obtained F1-score of 0.33. Additionally, we observed high variability of the results given different data splits. Thus, we restrict our analysis to identifying what factors are most important for performing classification. To obtain perfect sensitivity at least during the training time, we penalised false negative errors in the classification loss. This allowed us to identify the most important factors for predicting ERAF. The initial analysis suggested that out of 56 numerical variables only seven of them played an important role in predicting ERAF: TnT elevation, fluoroscopy time (min), LA Volume, PLT, TnT, CK-MB and CPK following CBA and TSH, INR before ablation (Fig. 1).

## Discussion

### Statistical analysis

In this study, the univariate analysis of the data identified that CK-MB and TnT after ablation with the TnT elevation were the independent predictors of ERAF following single CBA. Comorbidities such as diabetes mellitus and prediabetes were also significant ERAF predictors. It was noted that the patients treated with statins had a substantially increased risk of ERAF as opposed to those who did not have statins prescribed. Furthermore, the results in the univariate analysis indicated that Troponin T (*p* = 0.043), and CK-MB (*p* = 0.010) after ablation as well as TnT elevation (*p* = 0.044) and INR before ablation (*p* = 0.012) were associated with ERAF. This low myocardial biomarker release could be associated with lower lesion dimensions, and thus worse efficacy of the procedure. Therefore, the biomarker release could indirectly help to estimate cardiac lesion formation. The longer cryoenergy application time in the ERAF group could be the result of difficult PV anatomy and the poor cryoballoon contact.

Interestingly, several similar studies investigating the relation between myocardial injury biomarkers (CK, CK-MB, Tn) and ERAF have been conducted. For example, Kizilirmak et al. [11] reported that the elevation in CK, CK-MB, and Troponin I levels following CBA was associated with a lower ERAF rate as indicated in 3-month, 6-month and 9-month follow-up visits. Additionally, the increased levels of TnI were correlated with the median temperature. In the multivariate analysis only TnI elevation was the negative independent predictor of AF recurrence (specificity of 90.7% and a sensitivity of 64.3%; AUC 0.816, 95% CI 0.691–0.906). Another study carried out by Aksu et al. [12] showed that the lower postablation hsTnT level may predict an increased ERAF rate (*p* = 0.012). Lim et al. [13], similarly, found that the extent of TnT elevation as well as hsCRP predicted ERAF within 3 days after ablation but not following 3 months or 6 months. In the study of Casella et al. [14], by contrast, no significant association was observed between postprocedural TnI, CK-MB levels and ERAF in a 1-month, 3-month, 6-month and 12-month follow-up.

Our analysis has further revealed that the patients with diabetes or in a prediabetes condition had a significantly increased risk of ERAF. Other studies conducted so far have not been able to conclusively estimate the risk of ERAF in this group of patients given conflicting results. Nevertheless, in Chao’s et al. [15] research it was shown that patients with abnormal glucose metabolism had a greater AF recurrence rate after catheter ablation than those without it (18.5% vs 8.0%; *p* = 0.022). These findings were explained by changes in biatrial substrate properties with an intra-atrial conduction delay and a decreased voltage. It is worth noting that in Cai’s investigation [16] and D’Ascenzo’s et al. [17] meta analysis diabetes correlated with ERAF but not independently of other cardiovascular risk factors.

Apart from the correlation with diabetes, our results have also indicated that preablative intake of statins was more common in the group of patients with ERAF (*p* = 0.0007). The benefit of statin treatment proved to be significant in the prevention of postoperative AF and after electrical cardioversion. Nevertheless, previous studies on statin therapy designed to prevent AF recurrence after catheter ablation come to contradictory conclusions. In Sulaiman’s double blind placebo randomized trial it was found that an 80 mg dose of atorvastatin did not decrease ERAF risk in the first 3 months after the procedure [18]. In addition, in Dentali’s et al. [19] metaanalysis statins did not reduce ERAF risk after catheter ablation (RR 1.04; 95% CI 0.85–1.28, *p* = 0.71; *I*^2^ = 34%). It appears that larger randomized control trials are required to elucidate these results and obtain more conclusive data. Another finding relates to the pathophysiology of ERAF which depends on many factors. It has been demonstrated that ERAF within the first 3 months after ablation, called also “the blanking period,” is possibly associated with the inflammatory response, incomplete lesion healing, and autonomic nervous system (ANS) modulation [2]. Themistoclakis et al. [20] showed that longer AF duration, history of hypertension, left atrial enlargement, permanent AF were significantly associated with ERAF. Likewise, Bertaglia et al. [21] in their research pointed out that the structural heart disease and the lack of successful anatomical ablation of all targeted PV predicted early atrial tachyarrhythmias recurrence. Lee et al. [22], by contrast, showed that the presence of multiple AF foci could predict ERAF (*p* = 0.013; ratio = 2.24; 95% CI 1.18–4.25). Other research sources provide scoring systems with AF predicators which can foresee the short term AF recurrence risk (i.e. the APPLE, ALARMc, BASE-AF2) and long term recurrence risk (i.e. MB-LATER, CAAP-AF, PLAAF) following ablation [23].

However, it is still necessary to define the ERAF predictors which would allow for a better prevention system and a more suitable selection of patients susceptible to late AF recurrence. The statistical analysis and ML algorithms of so many parameters may facilitate such a selection prior the procedure, and thus increase its effectiveness. Identifying the patients with ERAF will help devise a better strategy to monitor the heart rhythm following 3 months since the CBA procedure and tailor further anticoagulant and antiarrhythmic treatment. Some factors related to predicting ERAF can also be directly linked to late and very late AF recurrence, which requires further research.

### Machine learning algorithms

The classification analysis confirmed the finding from the univariate analysis. TnT and CK-MB provide most information for predicting ERAF. Although classification modelling confirmed findings from the statistical analysis, all models provide unsatisfactory accuracy. This, in our view, is mostly due to the small data set. We believe that the performance of analysed algorithms can be greatly improved with a bigger size of the data which we hope to expand in the future.

## Conclusions

We showed that a simple biomarkers of myocardial injury such as CK-MB and TnT following CBA were significantly associated with ERAF. A greater myocardial biomarkers release has been associated with a better clinical outcome due to a more sustained PVI. Furthermore, diabetes mellitus, prediabetes and statin treatment were also classified as predictors of AF recurrence. Finally, ML algorithms confirmed the results of statistical analysis.

## Limitations

Our study was single centered with a relatively small number of patients. Further, a 24-h monitoring system was too short to capture all recurrence. Given that some patients could have asymptomatic AF recurrence, we might not have defined the exact number of ERAF.

Plasma levels of all biomarkers were measured at baseline and 24 h after CBA. Thus, we do not know the exact span of changes in the biomarkers level in the post-ablation follow-up. A long-term follow-up is needed to answer the question whether higher biomarkers of myocardial injury may be linked to a better clinical outcome. Finally, the main limitation of ML analysis was too small data set.
